# EFFECT OF HEARING INTERVENTION ON LONELINESS AND SOCIAL ISOLATION IN THE ACHIEVE STUDY

**DOI:** 10.1093/geroni/igad104.0532

**Published:** 2023-12-21

**Authors:** Alison Huang

**Affiliations:** Johns Hopkins Bloomberg School of Public Health, Baltimore, Maryland, United States

## Abstract

Social health interventions in older adults typically have small effect and limited large-scale implementation. Treating hearing loss is a potential intervention, but efficacy is unknown. This study presents baseline associations between hearing loss, loneliness, and social isolation as well as the 3-year effect of a hearing intervention on social health. The Aging and Cognitive Health Evaluation in Elders Study (ACHIEVE) randomized controlled trial (n=977) tests the effect of hearing intervention (provision of hearing aids/related technologies, counseling/education) vs. successful aging health education control intervention on loneliness (UCLA Loneliness Scale) and social network characteristics (Cohen Social Network Index) (pre-specified exploratory outcomes) among older adults with untreated hearing loss. Hearing loss was quantified by better ear, speech-frequency pure tone average (PTA). Speech-in-noise recognition and hearing-related quality of life were also measured. Baseline associations between hearing loss and social health outcomes are assessed by robust Poisson regression adjusted for demographic and health characteristics. Three-year hearing intervention effects are assessed by generalized linear mixed effects models. Participants were 70-84 years, 54% female, and 87% White. At baseline, better speech-in-noise recognition was associated with greater social network characteristics (e.g., larger social network size [IRR: 1.04, 95% CI: 1.00, 1.07]). Worse hearing-related quality of life was associated with higher prevalence of loneliness (PR: 1.15, 95% CI: 1.11, 1.20) and smaller social network size (IRR: 0.96, 95% CI: 0.93, 1.00). Hearing intervention effect results will be available prior to the conference (data collection ends early 2023). Hearing treatment could be an effective social health intervention with greater scalability.

